# The PRICE study (Protection Rest Ice Compression Elevation): design of a randomised controlled trial comparing standard versus cryokinetic ice applications in the management of acute ankle sprain [ISRCTN13903946]

**DOI:** 10.1186/1471-2474-8-125

**Published:** 2007-12-19

**Authors:** Chris M Bleakley, Seán O'Connor, Mark A Tully, Laurence G Rocke, Domnhall C MacAuley, Suzanne M McDonough

**Affiliations:** 1 Health & Rehabilitation Sciences Research Institute, University of Ulster, Jordanstown, Northern Ireland, UK; 2 Department of Emergency Medicine, Royal Victoria Hospital, Grosvenor Road, Belfast, Northern Ireland, UK; 3 Hillhead Family Practice, Belfast, Northern Ireland, UK

## Abstract

**Background:**

Cryotherapy (the application of ice for therapeutic purposes) is one of the most common treatment modalities employed in the immediate management of acute soft tissue injury. Despite its widespread clinical use, the precise physiological responses to therapeutic cooling have not been fully elucidated, and effective evidence-based treatment protocols are yet to be established. Intermittent ice applications are thought to exert a significant analgesic effect. This could facilitate earlier therapeutic exercise after injury, potentially allowing for a quicker return to activity. The primary aim of the forthcoming study is therefore to examine the safety and effectiveness of combining intermittent ice applications with periods of therapeutic exercise in the first week after an acute ankle sprain.

**Methods/Design:**

The study is a randomised controlled trial. 120 subjects with an acute grade I or grade II ankle sprain will be recruited from Accident & Emergency and a University based Sports Injury Clinic. Subjects will be randomised under strict double-blind conditions to either a standard cryotherapy (intermittent ice applications with compression) or cryokinetic treatment group (intermittent ice applications with compression and therapeutic exercise). After the first week, treatment will be standardised across groups. Assessor blinding will be maintained throughout the trial. Primary outcome will be function, assessed using the Lower Extremity Functional Scale (LEFS). Additional outcomes will include pain (10 cm Visual Analogue Scale), swelling (modified figure-of-eight method) and activity levels (*activ*PAL™ physical activity monitor, PAL Technologies, Glasgow, UK). Diagnostic Ultrasound (Episcan-1-200 high frequency ultrasound scanning system, Longport International Ltd, PA) will also be used to assess the degree of soft tissue injury. After baseline assessment subjects will be followed up at 1, 2, 3 & 4 weeks post injury. All data will be analysed using repeated measures analysis of co-variance (ANCOVA).

**Discussion:**

This paper describes the rationale and design of a randomised controlled trial which will examine the effectiveness of two different cryotherapy protocols in the early management of acute ankle sprain.

**Trial Registration:**

ISRCTN13903946

## Background

Ankle injuries represent one of the most commonly occurring musculoskeletal complaints. The vast majority of such injuries occur as a result of inversion trauma with the foot in some degree of plantar flexion and involve damage to the lateral structures of the ankle. Sprains of the lateral ankle ligaments are associated with significant costs [1] and account for an estimated 302,000 annual admissions to Accident & Emergency (A&E) Departments in the UK [2].

In addition to the immediate onset of pain, swelling and loss of joint motion, it has been reported that in 15 – 73% of cases, chronic ankle instability (CAI) with recurrent sprains and residual sensations of giving way may occur following lateral ankle sprain [3,4]. However, the precise etiology of CAI is unclear and as a consequence the optimal intervention for the management of acute ankle sprain is controversial. While a significant body of evidence supports the use of early functional treatment [5-7], there is little high quality research evidence to suggest which interventions best augment this treatment approach. Clinicians therefore continue to treat such injuries pragmatically, with current recommendations ranging from no intervention to physiotherapy referral, prophylactic bracing, or cast immobilisation [8-11].

Cryotherapy (the application of ice for therapeutic purposes) is a common treatment modality employed in the management of acute soft tissue injuries. Despite its widespread clinical use, the precise physiological responses to ice application have not been fully elucidated. Moreover, the rationale for its use at different stages of recovery is quite distinct. In the acute inflammatory phase after soft tissue injury, cryotherapy is thought to decrease oedema formation via induced vasoconstriction, and reduce secondary hypoxic damage by lowering the metabolic demand of injured tissues [12,13]. Cooling skin surface temperature to below approximately 15°C is also thought to exert a localised analgesic effect by inhibiting nerve conduction velocity [14,15]. Short periods of ice application have been used during the later, sub-acute phase of inflammation to produce a similar analgesic effect, thus facilitating earlier and more aggressive therapeutic exercise after muscle injury [16,17]. This combined use of cryotherapy and exercise has previously been termed *cryokinetics *[18]. Recent evidence has suggested that the addition of exercise to ice application is more effective than ice application alone after various soft tissue injuries, including acute ankle sprain [19]. However, by reducing the conduction velocity of other, non-nociceptive fibres, cold application may also have a number of deleterious effects, including reduced muscle torque [20]. This is of particular relevance if ice is to be applied in combination with therapeutic exercise in the early stages after an acute soft tissue injury. Such effects could lead to the development of altered neuromuscular control patterns and potentially, to an increased risk of re-injury. Conversely, other evidence has shown that ice application does not negatively affect myotatic reflex activity [21], joint position sense [22], plantar flexion torque [23] or more functional measures of agility [24,25]. Such conflicting findings may relate to the marked variation in cryotherapy protocols described in the literature, particularly in relation to the site, mode and duration of ice application. These factors, in addition to the level of subcutaneous fat, dictate the degree of superficial and deep tissue cooling, and therefore have a direct effect on the subsequent physiological response to cryotherapy [26].

Evidence from a large-scale systematic review suggested that intermittent ice applications of 10 minutes are most effective at reducing tissue temperature in both injured animal and healthy human models [27]. Such ice applications have been shown to reduce skin temperature to 5°C immediately after treatment [28]. A recent study by our research group also found that intermittent ice applications are more effective than continuous ice at reducing pain on activity after ankle sprain [29].

Given these findings, it seems a logical progression to examine if the analgesic effects of intermittent ice application can facilitate earlier therapeutic exercise, and subsequently improve clinical outcome following acute ankle sprain. The safety and effectiveness of incorporating therapeutic exercise with periods of intermittent ice application has not previously been examined in patients with acute soft tissue injury. The primary aim of the forthcoming trial is therefore to compare the effectiveness of standard intermittent versus cryokinetic ice applications in the management of acute grade I and grade II ankle sprains. In this manner we hope to contribute further to the existing evidence base in the area of acute soft tissue injury management.

## Methods/Design

**Figure 1:**
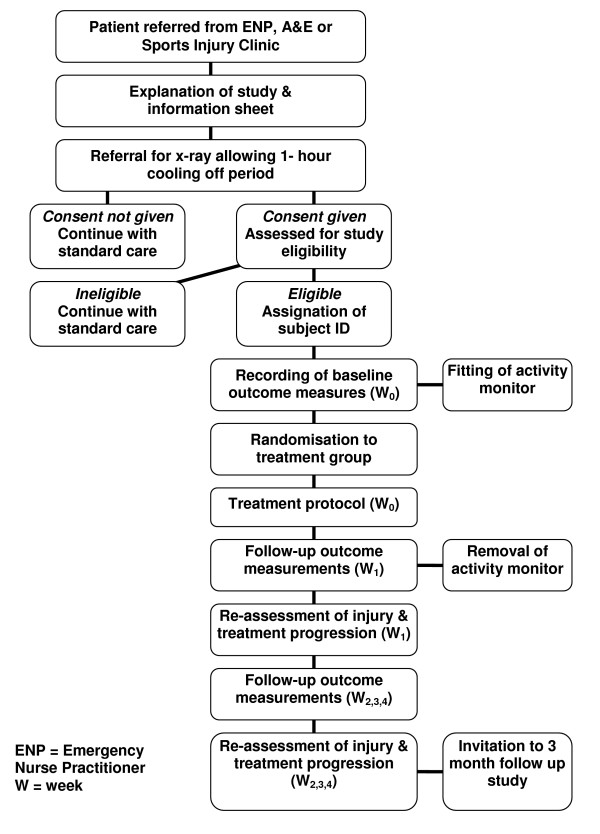
Summary of trial design.

The study is a randomised controlled trial (RCT). Figure 1 shows a brief summary of the trial design. The primary trial site will be the A&E Department of the Royal Victoria Hospital, Belfast, Northern Ireland. Subjects will also be recruited from the Sports Injury Clinic at the University of Ulster, Jordanstown, Northern Ireland. Following guidelines set out in the CONSORT statement [30], the number of patients assessed, randomised to treatment groups, who complete the study and who are included in the final analysis of the primary outcome shall be recorded during the trial. This will enable a participant flow diagram to be constructed. Ethical approval for the trial has been granted by The University of Ulster Research Ethics Committee [National Research Ethics Service reference number: 06/NIR03/148]. All patients who agree to take part will be required to give informed written consent prior to participation in the study.

### Study population

All patients between 16–65 years with an acute lateral ankle sprain (less than 1 week since injury) will be considered for participation in the study.

### Identification of potential subjects

Following initial assessment by an attending clinician (Emergency Nurse Practitioner, Physiotherapist or Doctor) patients with a suspected ankle sprain will be approached by a member of the research team and given a brief verbal explanation of the study. They will then be provided with a detailed study information sheet to consider while awaiting routine x-ray. This will allow for a standard 'cooling off' period of at least 1 hour.

### Inclusion/exclusion criteria

Patients with an acute grade I or grade II sprain will be included in the trial on the basis of a standard physical examination of the acutely injured ankle. As part of this assessment, the following demographic information will be recorded: date of birth, height, mass, gender, limb dominance and occupation. Activity level prior to injury will also be recorded and used to group patients according to whether they are from an athletic (defined as participating in high-intensity exercise for more than 90 minutes at least 3 times per week) or non-athletic population.

Patients will be excluded if any of the following criteria are present: Complete (Grade III) ankle ligament rupture (mechanical instability diagnosed by a positive anterior drawer or inversion stress test); Bony ankle injury (indicated by Ottawa ankle rules [31] or plain x-ray); Multiple injuries (e.g. other joint injury or fracture); Ankle sprain more than 1 week since injury; Any contraindication to cryotherapy including cryoglobinaemia, peripheral vascular disease or Raynaud's syndrome. Subjects will also be excluded if they are non-English speaking, have any condition which will affect understanding and communication, are under the influence of drugs/alcohol, or if there is no sufficient address given for follow-up.

### Baseline outcome measurements

Baseline recording of all outcome measures shall be carried out by the same researcher prior to randomisation.

#### Primary outcome measure

i) Subjective ankle function, assessed using the Lower Extremity Functional Scale (LEFS) [32]. The LEFS is an 80 point scale which has been shown to have excellent test-retest reliability (intraclass correlation coefficient [ICC] = 0.94, 95% confidence interval [CI] lower limit = 0.89). The scale has a potential error of ± 5.3 points (90% CI), with a minimal detectable change (MDC) and minimal clinically important difference (MICD) of 9 points (both 90% CI).

#### Secondary outcome measures

ii) Pain assessed using a 10 cm visual analogue scale, marked "no pain" at one end and "worst pain imaginable" at the other. This form of assessment is considered most appropriate because of its high level of repeatability when used serially on the same patient [33].

iii) Swelling assessed using a modified version of the figure-of-eight method [34]. High intra and inter-rater reliability has been reported using this technique (ICC = 0.99, 95% CI lower limit = 0.98), MDC = 9.6 mm (95% CI). To determine the degree of swelling, the mean value (of 2 measures) will be subtracted from the mean value of the uninjured ankle.

iv) Physical activity levels assessed using the *activ*PAL™ professional physical activity logger (PAL technologies, Glasgow, UK). Reported ICCs for inter-device reliability range from 0.79–0.97 (CIs not stated) [35]. The *activ*PAL™ unit (5 cm × 3 cm) will be worn on the thigh of the injured leg, for one week post injury. Time spent sitting, standing and walking under free living conditions will be compared between groups.

#### Tertiary outcome measure

**Figure 2:**
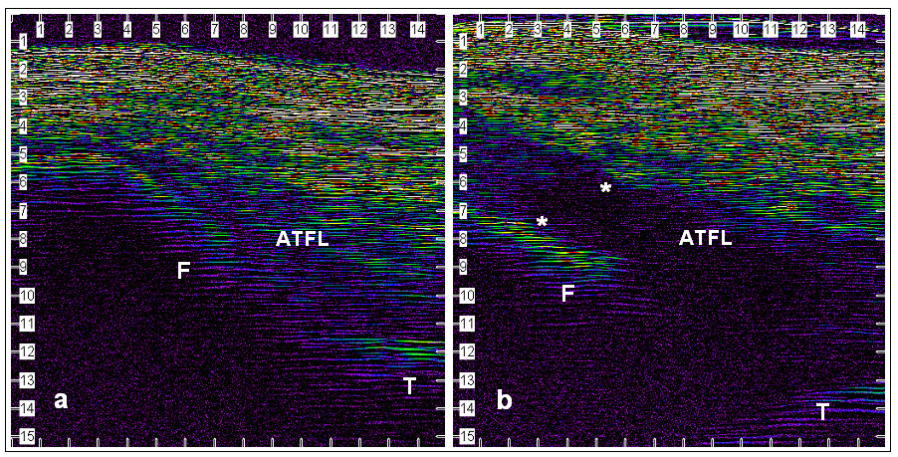
Ultrasound images of lateral ankle. (a) Un-injured control. (b) Grade I sprain 7 days post injury. The hypoechoic zone between the markers (*) indicates an area of soft tissue swelling. F = Fibula. T = Talus. ATFL = Anterior talofibular ligament. [Scale = mm]

v) Diagnostic ultrasound scanning (Episcan-1-200 high frequency ultrasound scanning system, Longport International Ltd, PA) will be used to examine the lateral structures of the injured and uninjured ankle. Images will be used to quantify the degree of soft tissue swelling and identify the relative positions of the fibula and talus (Figure 2). It is anticipated that this will provide a more accurate measure of swelling, and provide a useful tool to guide future clinical studies. For practical reasons, only those subjects who are recruited through the University Sports Injury Clinic will undergo ultrasound scanning.

### Treatment allocation

A statistician who will have no contact with the day to day running of the trial shall carry out all aspects of preparation for group randomisation. 184 randomisation envelopes will be produced in the following manner: 92 cards will be printed with **Group I (standard ice application)**. 92 cards will be printed with **Group II (cryokinetic ice application)**. Each card will be placed inside an opaque envelope with carbon paper on top. A randomisation sequence will then be generated using computer software. Stratified randomisation will be employed according to whether subjects are from an athletic (A) or non-athletic population (N). Separate block randomisation sequences will be produced for each stratum using an allocation ratio of 1:1 and a block size of 4 to ensure comparison groups are of approximate size. Envelopes for each stratum will be labeled sequentially (1A-92A & 1N-92N). Envelopes will then be sealed and signed across the seal. Following baseline assessment, these instructions, in sequentially numbered opaque sealed envelopes, will be used to assign sequentially enrolled subjects to one of the two treatment groups. Subjects shall therefore be randomised to treatment groups under strictly double-blind conditions. Before opening the envelope, the Research Physiotherapist will write the subjects unique identification number (stratification number & date of birth) on the outside of the envelope, transferring this information to the instruction card. At the end of the trial this will allow the Principle Investigator to check each card against the original randomisation lists and verify that all subjects received the treatment to which they were assigned.

### Treatment protocols

All subjects will receive an initial treatment, administered by the same Research Physiotherapist, in A&E or the University Sports Injury Clinic. Standard treatment (Group I) will consist of intermittent ice and compression only. Subjects will receive a 10 minute ice application. The ice pack will then be removed for 10 minutes before a further 10 minute ice application. This will then be followed by a further 10 minutes of rest (10 minutes ice/10 minutes rest/10 minutes ice/10 minutes rest). Cryokinetic treatment (Group II) will consist of intermittent ice and compression with therapeutic exercise. Subjects will receive a 10 minute ice application. The ice pack will then be removed and the subject will perform 10 minutes of therapeutic exercise. This will be followed by another 10 minute ice application and a further 10 minutes of therapeutic exercise (10 minutes ice/10 minutes exercise/10 minutes ice/10 minutes exercise). Subjects will be responsible for ice pack preparation and self administering subsequent treatments at home (3 times per day for the first week after injury). Compliance with treatment and analgesic consumption will be monitored by the use of a treatment diary. Standard advice regarding general mobilisation exercises and weight bearing will also be given to both groups according to routine A&E practice.

### Cryotherapy

**Figure 3:**
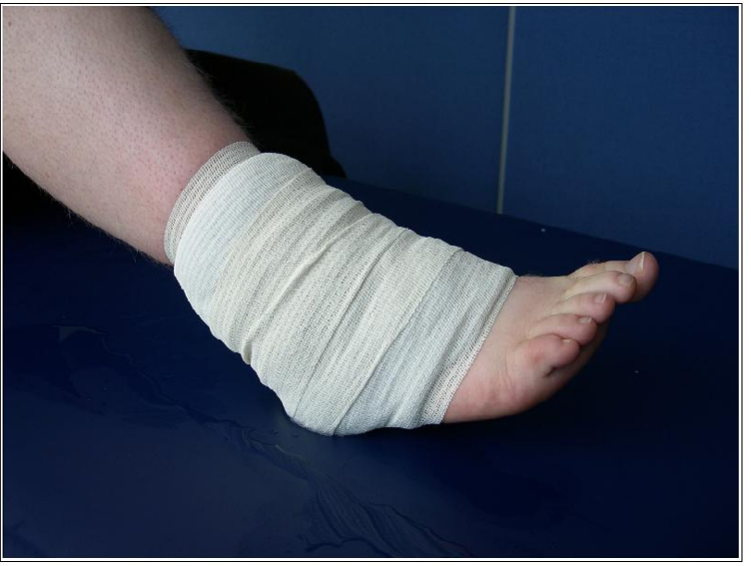
Method of ice pack application.

Mode of cryotherapy will be standardised across groups (melting iced water [0°C] in a standard sized pack). Clear plastic commercial ice cube bags (17 cm × 28 cm) will be completely filled with water and frozen. Before application, ice packs will be held under hot running water for 30 seconds and wrapped in a single layer of towelling (moistened until just dripping wet). The packs will then be placed over the lateral aspect of the ankle joint, covering an area from the Achilles tendon to the anterior tibialis muscle, with the approximate center of the pack overlying the anterior talofibular ligament (ATFL). Compression will be applied over the pack using 8 cm cohesive bandaging with approximately 5–6 cm of stretch (Figure 3). Timing of the cryotherapy protocol will begin as soon as the compression bandage is in place. A standard verbal explanation and step by step written instructions of the correct procedure for ice pack preparation and application will be given. All necessary equipment will be provided.

### Exercise

Subjects in group II will be provided with verbal and written instructions, and a DVD demonstrating each of the additional exercises. The exercise component (Table 1) has been adapted from a standard written protocol [36] and is designed to take approximatly 10 minutes to complete.

**Table 1:** Therapeutic exercises for treatment group II

**Exercise**	**Repetitions**	**Time (Sec)**
Circumduction (clockwise/anticlockwise)	20	60
Active pf/df	20	60
Static ev/in/pf/df (with 10 second hold)	5 of each	300
Heel slides (with lower limb triple extension)	30	120
Static calf stretch (with 30 second hold)	3	60

pf = plantar flexion. df = dorsi flexion. ev = eversion. in = inversion.

### Follow-up procedure & blinding

At week 1 post injury, function, pain and swelling (outcome measures i, ii, and iii) will be re-assessed by the same researcher who shall remain blinded to group allocation throughout the trial. The activity monitor will also be removed for data collection at this stage (outcome measure iv). Subjects will hand their treatment diaries to the Research Physiotherapist who will re-assess the injured ankle. Follow-up ultrasound images (outcome measure v) shall be taken in those subjects recruited through the University Sports Injury Clinic. After week 1, individual treatment will be progressed in both groups according to clinical need, but will follow a standarised protocol consisting of early, intermediate and advanced stage muscle strengthening, proprioceptive and functional exercises. Subsequent follow-ups shall take place at weeks 2, 3 and 4. Researcher blinding is described in Table 2.

**Table 2:** Blinding of each researcher during trial.

Researcher	Role	Blinding status
RP1	Recruitment, assessment, randomisation and treatment	Unblinded
BA	All baseline and follow-up recording of function, pain, swelling and activity levels (outcome measures i, ii, iii, iv)	Blinded to group allocation
RP2	US images (outcome measure v)	Blinded to group allocation
IR	Interpretation of US images (outcome measure v)	Blinded to group allocation

RP = Research Physiotherapist. BA = Blinded Assessor. US = Ultrasound. IR = Independent Researcher.

### Additional follow up study

In a sub-section to the main trial, longer term function and ankle muscle strength will be examined in a sample of study subjects at 3 months post-injury. While chronic instability and recurrent injuries are a frequent complication following an initial sprain, the precise reasons why such injuries tend to reoccur are unclear at present, and as a consequence rehabilitation may be problematic. It has been suggested that specific evertor muscle strength deficits might be a significant factor in the pathogenesis of CAI [37,38]. However, other evidence does not support this contention [39,40]. Previous research has examined conventional eccentric to concentric ratios of individual muscle groups, or concentric evertor to invertor ratios [39-42]. Since dynamic joint stabilization is achieved by co-contraction of the muscles surrounding the joint, it might be more appropriate to divide the eccentric moment of the antagonist by the concentric moment of the agonist [43]. Examination of such dynamic, reciprocal muscle-group ratios represents an alternative approach to the assessment of muscle strength deficits and imbalance following ankle sprain [44]. Here, muscle strength will be assessed using a KinCom 500H isokinetic dynamometer (Chattecx Corp., Hixson, TN). Isokinetic dynamometers provide an accommodating resistance throughout full range of motion, and have been shown to provide a safe and reliable measure of ankle strength [45]. Eccentric and concentric peak torque values for eversion, inversion, plantar flexion and dorsi flexion will be recorded at a velocity of 60° sec^-1^. Raw data will be normalised for body weight before dynamic ratios are calculated and compared to the un-injured (control) ankle. For example, the eccentric eversion to concentric inversion (eE/cI) ratio for a subject with a body weight (BW) of 88 kg is calculated in the following way: If eE = 81 Newton metres (N.m) (eE/BW = 0.92 N.m/kg) and cI = 92 N.m (cI/BW = 1.15 N.m/Kg), the eE/cI ratio would therefore be 0.92/1.15 = 0.80.

### Sample size

**Figure 4:**
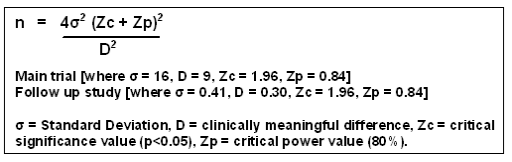
Formula used for sample size calculations.

Using data from a previous published study [32] an appropriate sample size for the main trial has been determined using the formula shown in Figure 4[46]. Based on a clinically meaningful difference for the primary outcome measure of 9 points, and allowing for a 10% attrition rate, a minimum number of 60 subjects will be required for each treatment group (assuming 80% power and a 0.05 alpha value).

Using the same formula, a sample size calculation has also been carried out to determine the number of subjects required in the 3 month follow up study. This calculation is based on published data [47] and a clinically meaningful difference between ankle muscle strength ratios of approximately 30%. 30 subjects will be required with the un-injured ankle acting as the control.

### Statistical analysis

Analysis will be on an intention to treat basis. Data will be analysed using SPSS (Windows Version 14.0). Assuming data is from a normal distribution, descriptive statistics will be performed to produce standard deviations (SD), standard errors of the mean (SEM) and 95% confidence intervals (CI). For all outcome measures, a within subject repeated measures analysis of covariance (ANCOVA) will be calculated to determine significant changes over time between groups. Treatment group (two levels: standard and cryokinetic ice application) will be the between subjects factor. Time (four levels: week 1, 2, 3 & 4) will be the within subject factor, with baseline values (week 0) used as the covariate. Where Mauchley's test reveals the assumption of sphericity had been violated the Greenhouse-Geisser epsilon procedure will be carried out to adjust the degrees of freedom accordingly. For all analysis Tukey's test will be used to make *post hoc *adjustments for multiple comparisons. If any significant interactions are detected between treatment group and time point, a univariate analysis of covariance will be used to indicate the time point at which significant differences are found. If required, analyses will be undertaken using the expectation maximisation algorithm method to impute missing values [48]. A number needed to treat (NNT) analysis will also be carried out. For the 3 month follow-up study, a 2 × 2 ANOVA will be calculated to determine if any significant differences exist between isokinetic ratios of subjects injured and un-injured (control) ankles. The level of significance for all tests will be set at p < 0.05.

## Discussion

Here we have described the rationale and design of a randomised controlled trial comparing standard intermittent versus cryokintic ice applications in the early management of acute grade I and grade II ankle sprain. Although such injuries are often regarded as being fairly innocuous, recurrent sprains and sensations of instability are a frequent sequelae of lateral ankle sprain. Perhaps this is not surprising, given the complexity of the ankle joint and the uncertainty regarding the precise aetiology of chronic ankle instability. However, it may be that current treatment recommendations are insufficient. More intensive initial treatment and advice on potential complications may help to reduce the incidence and associated costs of long-term symptoms after an initial sprain. Intermittent, ten minute periods of ice application and therapeutic exercise in the early stages after injury may represent a simple and cost effective intervention for both athletic and non-athletic populations. However, high quality randomised controlled trials are first required in order to examine the effectiveness of such interventions.

The difficulties of conducting research in an acute setting have previously been highlighted [49]. The process of ensuring all potential patients are assessed for study eligibility will be aided by the researchers being based in A&E on a day to day basis, by working closely with Emergency Nurse Practitioners responsible for triage assessment, and by providing regular trial updates for other relevant clinical staff. Trial profile shall be maintained by placing posters in A&E and by regularly updating media sources including the Hospital and University intranet.

Recruitment will begin in July 2007 and it is anticipated that all data collection will be completed by July 2008. Results of the trial will be disseminated through publication in relevant peer-reviewed journals and conference proceedings.

## Competing interests

The author(s) declare that they have no competing interests.

## Authors' contributions

CMB wrote the original protocol, secured funding and will be responsible for ultrasound imaging and the overall management of the trial. SOC contributed to the development of the protocol, wrote this manuscript and will be responsible for subject recruitment and treatment during the trial. MAT contributed to the development of the protocol and will be responsible for data handling during the trial. LGR will act as co-principle investigator and will be responsible for the overall management of the clinical setting in which the research is to take place. DCM wrote the original protocol and helped secure funding. SMD wrote the original protocol, secured funding and will act as co-principle investigator. CMB, SOC, MAT and SMD will be responsible for data analysis and interpretation of results. All authors have contributed to and approved the final version of this manuscript.

## Pre-publication history

The pre-publication history for this paper can be accessed here:


